# Dual-energy CT angiography in detecting underlying causes of intracerebral hemorrhage: an observational cohort study

**DOI:** 10.1007/s00234-024-03473-1

**Published:** 2024-10-25

**Authors:** Michaël T. J. Peeters, Alida A. Postma, Robert J. van Oostenbrugge, Wouter J.P. Henneman, Julie Staals

**Affiliations:** 1 Department of Neurology, School for Cardiovascular Diseases Maastricht (CARIM), Maastricht University Medical Center, P. Debyelaan 25, Maastricht, 6229 HX The Netherlands; 2 Department of Radiology and Nuclear Medicine, Maastricht University Medical Center, Maastricht, Netherlands; 3 Mental Health and Neuroscience research institute (MHeNs), Faculty of Health, Medicine and Life Sciences, Maastricht University, Maastricht, Netherlands

**Keywords:** Dual-energy computed tomography angiography, Intracerebral hemorrhage, Etiology

## Abstract

**Background:**

CT angiography (CTA) is often used to detect underlying causes of acute intracerebral hemorrhage (ICH). Dual-energy CT (DECT) is able to distinguish materials with similar attenuation but different compositions, such as hemorrhage and contrast. We aimed to evaluate the diagnostic yield of DECT angiography (DECTA), compared to conventional CTA in detecting underlying ICH causes.

**Methods:**

All non-traumatic ICH patients who underwent DECTA (both arterial as well as delayed venous phase) at our center between January 2014 and February 2020 were analyzed. Conventional CTA acquisitions were reconstructed (‘merged’) from DECTA data. Structural ICH causes were assessed on both reconstructed conventional CTA and DECTA. The final diagnosis was based on all available diagnostic and clinical findings during one-year follow up.

**Results:**

Of 206 included ICH patients, 30 (14.6%) had an underlying cause as final diagnosis. Conventional CTA showed a cause in 24 patients (11.7%), DECTA in 32 (15.5%). Both false positive and false negative findings occurred more frequently on conventional CTA. DECTA detected neoplastic ICH in all seven patients with a definite neoplastic ICH diagnosis, whereas conventional CTA only detected four of these cases. Both developmental venous anomalies (DVA) and cerebral venous sinus thrombosis (CVST) were more frequently seen on DECTA. Arteriovenous malformations and aneurysms were detected equally on both imaging modalities.

**Conclusions:**

Performing DECTA at clinical presentation of ICH may be of additional diagnostic value in the early detection of underlying causes, especially neoplasms, CVST and DVAs.

**Supplementary Information:**

The online version contains supplementary material available at 10.1007/s00234-024-03473-1.

## Introduction

Computed tomography angiography (CTA) is often used as initial screening tool in the acute phase of non-traumatic ICH to detect underlying causes. A 2014 Cochrane review reported 95% sensitivity and 99% specificity for detecting intracranial vascular malformations [1]. However, a more recent study in a selected population having a high pre-test probability of harboring a vascular abnormality (by excluding older hypertensive patients with basal ganglia ICH), showed CTA to have a more modest sensitivity and specificity of 74% and 91% respectively, for detecting macrovascular ICH causes [2]. The diagnostic accuracy of CTA for detecting other, less frequently encountered secondary ICH causes such as hemorrhagic neoplasms, cerebral venous sinus thrombosis (CVST), cavernomas or developmental venous anomalies (DVAs), is unknown. These causes are often considered together with more common macrovascular causes or are disregarded in studies [2–5]. It seems likely that CTA has a lower diagnostic yield for detecting these ICH causes. For example, it is difficult to visually distinguish iodine enhancement from hyperdense hemorrhage in hemorrhagic neoplasms, and the detection of CVST can be complicated by the impeding arterial contrast phase timing and appearance of dense thrombi which could be mistaken for vascular enhancement.

Dual-energy computed tomography (DECT) is a promising CT technique which is increasingly being applied in practice, and is readily available in the acute setting. By simultaneously using high – and low-peak voltage acquisitions and applying a post processing algorithm, different materials can be separately characterized due to their differential attenuation at two different energy levels. In acute ICH, DECT can be used to differentiate hemorrhage (blood products) from iodinated contrast agent [6]. We previously showed that the diagnostic accuracy of the spot sign, a predictor for hematoma expansion in ICH, is higher on DECT angiography (DECTA) when compared to single-energy imaging (CTA) [7]. Studies on DECTA in structural ICH causes are scarce, but have shown that DECTA can better detect lesions near osseous structures such as arteriovenous fistulas (AVF) and aneurysms, when compared to single energy CTA [8, 9]. Furthermore, a small study found DECTA, when applied at a mean of 4 days after symptom onset, to have a higher diagnostic accuracy in differentiating intracranial tumor bleeding from non-tumor bleeding, when compared to conventional contrast-enhanced CT [10]. Applying DECTA directly at presentation in acute ICH could therefore be more informative than conventional CTA for detecting vascular and structural underlying lesions.

This study aims to evaluate and compare the diagnostic yield of post processed DECTA to conventional CTA in the detection of underlying ICH causes.

## Materials and methods

### Study design and patient selection

All imaging confirmed adult non-traumatic ICH patients presenting at the Maastricht University Medical Center (MUMC+), the Netherlands, between January 1st 2014 and February 29th 2020, who underwent DECTA were prospectively included. The medical ethical committee of the MUMC + approved the study and written informed consent for the collection of clinical and radiological data was obtained if possible, but waived in case a patient died before consent or when the patient was incapacitated by the stroke and no legal representative was available.

Patients with traumatic ICH, hemorrhagic transformation of ischemic stroke and non-parenchymal hemorrhage (e.g. primary intraventricular hemorrhage, epidural, subdural and primary subarachnoid hemorrhage) were not included. Patients who did not undergo DECTA, whose DECTA datasets were incomplete or in whom the delay between first brain CT and DECTA exceeded 24 h were excluded, as were patients with a previously known vascular - or structural cerebral lesion explaining their hemorrhage.

### Image acquisition and analysis

As part of standard care in ICH patients, non-contrast brain CT (NCCT) was followed by DECTA using a dual-source CT-system (Somatom Definition Flash or third generation Somatom Definition Force; Siemens Healthcare, Forcheim Germany). Patients received 90 ml intravenous contrast media (Ultravist 300 mg/ml, Bayer Healthcare, Leverkusen, Germany) with a flow of 5 ml/sec. The DECTA protocol covered both arterial phase imaging, based on timing after 10 cc testbolus, as well as 80-second delayed (venous) phase imaging. Imaging parameters of NCCT and DECT angiography are described elsewhere [7].

**Fig. 1:**
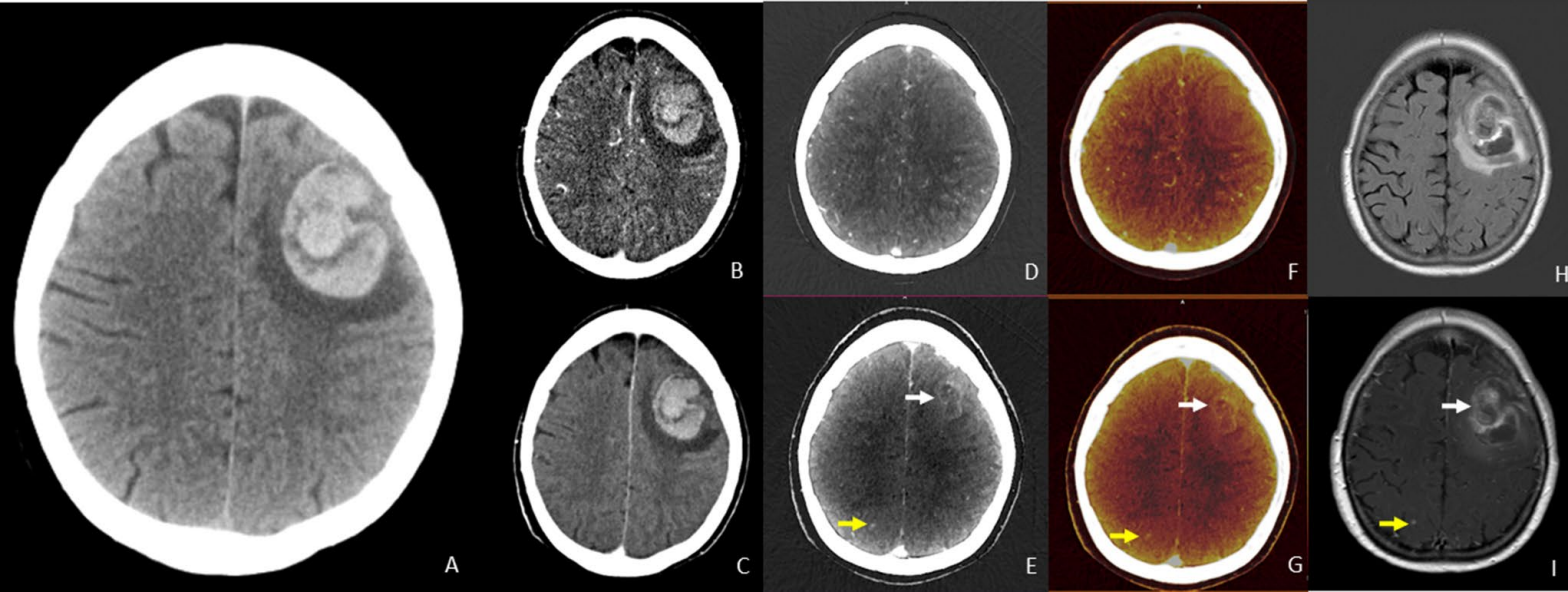
Left frontal ICH with underlying metastasis of a non-small-cell lung carcinoma in a 69-year old patient who presented with acute headache and aphasia. (**A**) The non-contrast CT shows left frontal intracerebral hemorrhage with surrounding edema. (**B**-**C**) Conventional-like CTA arterial and delayed phase images. (**D**-**F**) Arterial phase iodine – and fusion images. (**E**-**G**) Delayed phase iodine and fusion images show a ring-enhancing lesion (white arrow) suspected for metastasis, which was confirmed on fluid attenuated inversion recovery (FLAIR) and contrast enhanced MR images (**H**-**I**). The yellow arrow points to a contralateral enhancing lesion on delayed phase iodine and delayed phase fusion imaging as well as MR imaging, indicating a small contralateral cerebral metastasis.

Conventional CTA images simulate single-energy acquisitions at 120kVp, and were generated by linear blending of the high – and low energy datasets with a weighted average of 0.6. These reconstructed conventional CTA images were generated for both the arterial and delayed phase [11]. Dual-energy post-processing was performed using SyngoVia (Siemens Healthcare) to generate iodine maps (arterial iodine and delayed iodine) using a three material decomposition algorithm “Brain Haemorrhage” [12–14]. Fusion images (arterial fusion and delayed fusion) were created by registration and overlaying of the iodine maps with the mixed images. Figure 1 shows an example of the different imaging datasets.

Thirty imaging sets were reviewed by two independent neuroradiologists (A.P and W.H.) in order to assess inter-rater-reliability using Cohen’s kappa (κ), which was moderate with a κ of 0.554. Here-after all NCCT and DECTA datasets were reviewed retrospectively by a single experienced neuroradiologist (A.P.) blinded to clinical data and having > 10 years of experience. First, the conventional CTA images (arterial phase and delayed phase) were reviewed, then, the DECTA images (iodine arterial phase, fusion arterial phase, iodine delayed phase and fusion delayed phase) were reviewed consecutively for the presence of underlying pathology. Underlying structural causes of acute ICH were categorized as follows: arteriovenous malformation (AVM) / dural arteriovenous fistula (DAVF), aneurysm, neoplasm, cerebral venous sinus thrombosis (including isolated cortical venous thrombosis) and developmental venous anomaly (DVA). DVA were only considered as possible cause for ICH when associated to the ICH location, possibly pointing to a coexisting and associated cavernous malformation.

### Clinical data

Medical records were reviewed for patient age, sex, known hypertension, anticoagulant use, time between last seen well and DECTA imaging, Glascow coma scale (GCS) score on admission and in-hospital mortality. If present, reports of magnetic resonance imaging (MRI), MR angiography (MRA), digital subtraction angiography (DSA) as well as intraoperative and pathology findings were collected up to one year after the ICH.

### Diagnostic yield of imaging modalities and comparison to reference standard

Final diagnosis was the best available evidence from all diagnostic findings during 1-year follow up. The following causes of ICH were defined as reference standard: ICH related to hypertensive arteriopathy, when there was a history of hypertension or use of antihypertensive medication, hypertensive ICH location (basal ganglia, thalamus, pontine or cerebellum), and no coagulopathy or structural cause. Other defined causes include CAA, when fulfilling the modified Boston criteria for definite or probable CAA [15], coagulopathy related ICH (when no structural cause was found and platelet count on admission was < 50 × 10^9^ per liter of blood, INR > 3.0, aPTT > 80 s or in case of therapeutic direct oral anticoagulant (DOAC) use), vascular malformations (both AVM and DAVF), CVST (including isolated cortical venous thrombosis), neoplasms (both primary brain tumors and metastasis), aneurysmatic parenchymal ICH, cavernoma or DVA spatially related to ICH location, other (e.g. drugs/vasculitis/septic embolism) or unknown (when not fulfilling any of the categories above). In case no confirmatory examination was performed, if a vascular or structural etiology was identified on the conventional like CTA or DECTA, it was considered the final diagnosis. When there was doubt about the underlying pathology, or in case of contradictory findings on imaging, consensus was reached between one neuroradiologist (A.P), one vascular neurologist (J.S.) and one resident neurology (M.P.).

### Statistical analysis

Statistical analysis was performed using SPSS for Windows (version 25.0, IBM Corp., Amonk, NY, USA). Data are presented as mean and standard deviation for normally distributed continuous variables, median with interquartile range for non-normally distributed continuous variables and frequency with percentages for categorical variables.

## Results

A total of 339 non-traumatic ICH patients presented to our emergency department between January 1st 2014 and march 1st 2020. Supplementary Table 1 illustrates the flow chart of the study. We excluded 133 patients (39.3%): in 96 patients DECTA was not performed. A very poor prognosis on presentation and/or diminished kidney function were the most frequently reported reasons for not performing DECTA imaging. Three patients had a known underlying neoplastic or vascular lesion explaining their hemorrhage. DECTA source data of 34 patients were unable to be post-processed using the modern post-processing algorithm. These cases were therefore excluded from analysis. The remaining 206 patients (60.7%) were included in the study.

### Baseline characteristics

Of the included 206 patients, 83 were female (41.3%). Median age was 72 years (IQR 21.0 years) and 63 patients (30.6%) died during hospital admission. Hypertension was present in 131 patients (63.6%). Oral anticoagulants were used by 47 patients (28.8%). Median time between onset or last-seen-well (LSW) and DECTA imaging was 5.0 h (IQR 15.0 h). At one-year follow up, MRI and/or MRA had been performed in 75 patients (36.4%), Digital Substraction Angiography (DSA) in 8 patients (3.9%), and surgery results or histology was available in 17 patients (8.3%). Baseline characteristics are presented in Table 1.

**Table 1:** Baseline characteristics of included patients

Clinical	*N* = 206
Age, median [IQR], y	72 [21]
Male sex, n (%)	123 (59.7)
History of hypertension, n (%)	131 (63.6)
Anticoagulant use, n (%)	47 (22.8)
GCS, median [IQR]	14 [3]
In-hospital mortality, n (%)	63 (30.6)
**Imaging**	
ICH location, n (%)	
Lobar	104 (50.5)
Basal ganglia/thalamic	80 (38.8)
Infratentorial	18 (8.7)
Multifocal	4 (1.9)
IVH, n (%)	80 (38.8)
Time from LSW to imaging (DECT angiography), median [IQR], hours (*n* = 202 patients)	5 [15]
MRI/MRA performed, n (%)	75 (36.4)
DSA performed, n (%)	8 (3.9)
Available histology/surgical reports	17 (8.3)

SD indicates standard deviation; ICH, intracerebral hemorrhage; GCS, Glasgow Coma Scale; IQR, interquartile range; LSW, last seen well; DECT, dual-energy computed tomography; CT, computed tomography; IVH, intraventricular hemorrhage; DSA; digital subtraction angiography, MRI, magnetic resonance imaging and MRA, magnetic resonance angiography

### Final diagnosis at one year and diagnostic yield of conventional like CTA and DECTA imaging

A secondary (structural or vascular) ICH diagnosis was made in 30 patients (14.6%) at one year follow-up. Cavernomas or DVAs spatially related to the ICH location were the most frequently encountered lesions (*n* = 10, 4.9%), followed by neoplasms (*n* = 7, 3.4%), AVM/DAVF (*n* = 6, 2.9%), aneurysms (*n* = 4, 1.9%) and CVST (*n* = 3, 1.5%). Hypertensive arteriopathy (33.1%) was considered the most common non-structural cause of ICH, followed by coagulopathy related ICH (14.6%) and CAA (11.6%). Patients not fulfilling any of the predefined categories were labelled as unknown ICH cause (*n* = 52 patients, 25.2%). Final ICH diagnoses at one year follow-up are presented in Table 2.

**Table 2:** Underlying ICH causes, final ICH diagnosis at one year follow-up

Cause of ICH	No (%) of patients (*n* = 206)
ICH associated with hypertensive arteriopathy	68 (33.1)
Unknown	52 (25.2)
Coagulopathy related ICH	30 (14.6)
Probable/definite CAA	24 (11.6)
Cavernoma or DVA spatially related to hematoma	10 (4.9)
Neoplasm	7 (3.4)
Vascular malformation (AVM or DAVF)	6 (2.9)
Aneurysm	4 (1.9)
CVST/isolated cortical vein thrombosis	3 (1.5)
Other (e.g. drugs, vasculitis, RCVS)	2 (1.0)

ICH indicates intracerebral hemorrhage; DVA, developmental venous anomaly; AVM, arteriovenous malformation; DAVF, dural arteriovenous fistel; CVST, cerebral venous sinus thrombosis; CAA, cerebral amyloid angiopathy and RCVS, reversible cerebral vasoconstriction syndrome

**Fig. 2:**
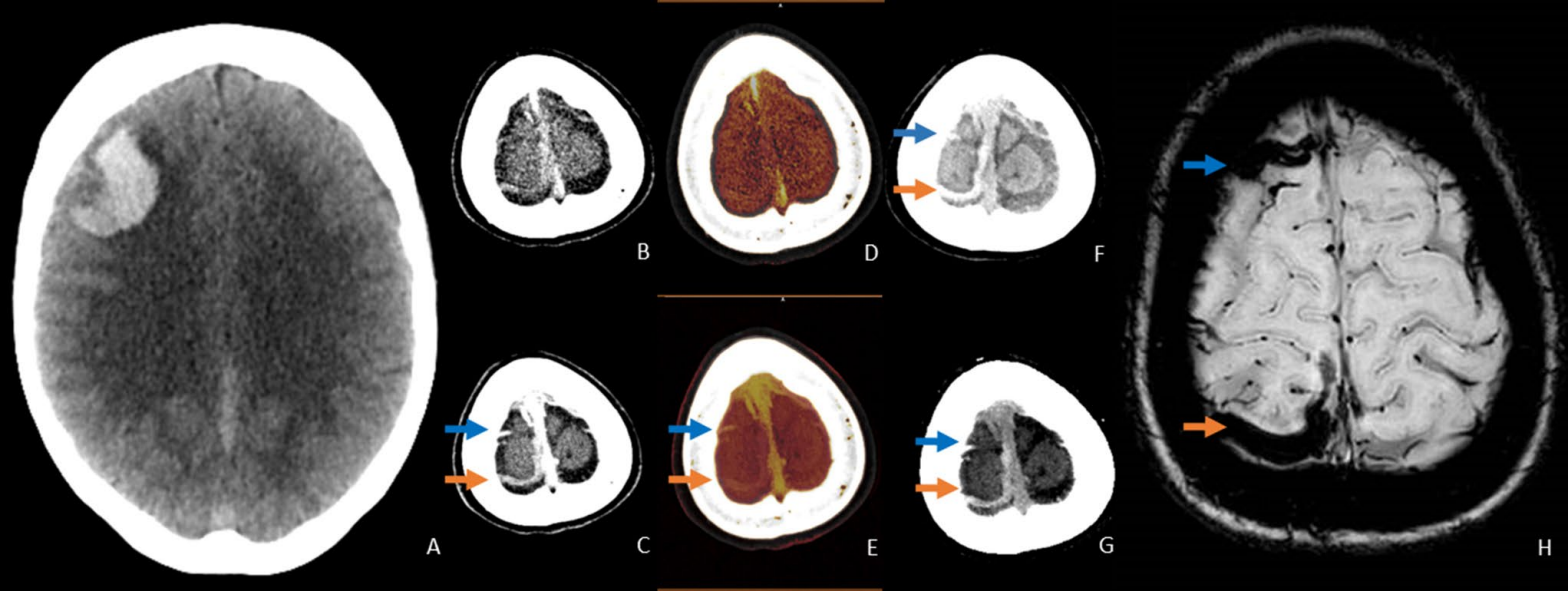
Right frontal ICH based on an isolated cortical vein thrombosis in a 48-year-old woman who presented with an epileptic seizure following a two-weak history of subacute headache. (**A**) Non-contrast CT shows a right frontal lobar ICH. (**B**) Conventional CTA and (**C**) conventional delayed phase imaging (simulating single energy CTV) show two dense cortical venous vessels (frontal and parietal; the blue and orange arrow respectively) which seem to be unobstructed (having contrast enhancement without apparent filling defect in image **C**). As expected, DECT arterial phase fusion imaging (**D**) does not show any contrast filling in the cortical veins. However, the delayed phase fusion images (**E**) show a diminished (blue arrow) and absent (orange arrow) filling in these vessels, indicating local thrombosis. (**F**) The non-contrast CT images and virtual non-contrast images (**G**) show the same dense cortical venous vessels, consistent with dense thrombus. Axial susceptibility weighted MR imaging the day after (**H**) confirm the local thrombosis in both cortical vessels. This example demonstrates the difficulty of differentiating the density of thrombus from contrast-staining on conventional-like delayed phase images in isolated cortical vein thrombosis, with improved visibility on DECTA reconstructions.

Table 3 Shows the diagnostic yield - and error of conventional CTA and DECTA. Conventional CTA showed an underlying ICH cause in 24 patients (11.7%) of whom six were false positive (2.9%). DECTA imaging showed an underlying cause in 32 patients (15.5%) of whom four were false-positive (1.9%). In 12 cases, findings were false negative on conventional CTA (5.8%), compared to two false negative findings on DECTA (1.0%). The diagnostic yield was greater for DECTA compared to conventional CTA for detecting neoplastic ICH, ICH related to CVST or isolated cortical venous thrombosis, as well as ICH associated DVA and/or cavernoma. DECTA and conventional CTA were equally effective in establishing the diagnosis of aneurysmatic parenchymal ICH and ICH related to an underlying AVM and/or DAVF. Figure 1 shows an example of a ICH due to an underlying metastasis, as shown on DECTA, which was not visible on conventional CTA. Three patients were diagnosed with CVST, of whom two had an isolated cortical vein thrombosis. The conventional CTA showed CVST in one case, DECTA aided in the demonstration of two cases of isolated cortical vein thrombosis with associated ICH. Figure 2 shows an example of the imaging of isolated cortical vein thrombosis on both imaging modalities

**Table 3:** Diagnostic yield and error of (DE)CTA in detecting underlying ICH pathology

Final diagnosis of vascular or neoplastic lesion	DECTA	Conventional CTA
**Total**		
30	28 - 2 false negative - 4 false positive	18 - 12 false negative - 6 false positive
**AVM/AVF**		
6 5 MRA or DSA confirmed 1 based on convincing CTA and DECTA	6 - No false negative - 1 false positive	6 - No false negative - 2 false positive
**CVST**		
3 2 MR confirmed 1 based on clinical grounds and convincing DECTA	3 - No false negative - No false positive	1 - 2 false negative - No false positive
**Aneurysm**		
4	4	4
1 MRA/DSA confirmed	- No false negative - No false positive	- No false negative - No false positive
3 based on convincing CTA and DECTA		
**Neoplasms**		
7 5 MR confirmed 2 based on clinical grounds	7 - No false negative - 3 false positive	4 - 3 false negative - No false positive
**DVA/cavernoma**		
10 6 MR confirmed 4 based on convincing CTA and DECTA	8 - 2 false negative - No false positive	3 - 7 false negative - 4 false positive

ICH indicates intracerebral hemorrhage; DVA, developmental venous anomaly; AVM, arteriovenous malformation; DAVF, dural arteriovenous fistel; CVST, cerebral venous sinus thrombosis; (DE)CTA, (Dual-energy) computed tomography angiography; MR, magnetic resonance; DSA, Digital subtraction angiography

## Discussion

In this study we show that DECTA has a higher diagnostic yield in acute ICH for detecting underlying neoplasms, DVAs and CVST when compared to conventional CTA. Findings were similar on both imaging modalities for other underlying ICH causes, including AVMs, DAVFs and aneurysms. Both false-positive and false-negative results occurred more frequently on conventional CTA. There were no cases in which a secondary or structural ICH cause was found on conventional CTA, but not on DECTA.

Primary ICH, originating from hypertensive arteriopathy or CAA, accounts for 78–88% of all ICH, and the remaining 12–22% of ICH have a secondary cause, including coagulopathy-related ICH and structural (vascular or neoplastic) causes [16]. In our cohort, 26.7% of all patients had a secondary ICH cause. The high coagulopathy associated ICH incidence in the Netherlands [17] and inclusion of DVAs as secondary ICH cause might explain this high percentage of secondary ICH.

In line with numbers found in literature, a neoplastic cause of ICH was found in 3.4% of our study population [18–20]. DECTA at clinical presentation detected an underlying malignancy in all seven patients (3.4%) carrying a definite neoplastic ICH diagnosis after one year. Conventional CTA detected only four of these cases. A single other study compared DECT to enhanced average (EA) imaging (equivalent to post contrast imaging) in order to differentiate neoplasm-related bleeding from non-neoplasm bleeding. In this study, DECT and EA imaging were simultaneously performed in 56 non-hypertensive patients with unknown origin ICH after a mean interval of 4.3 days between symptom onset and imaging. An underlying neoplasm was diagnosed in 17 patients (30.4%), which is remarkably high. Compared to EA imaging, DECT imaging had a higher sensitivity and specificity for detecting underlying neoplasms [10]. We show that DECTA retains its diagnostic superiority over conventional CTA for detecting neoplastic ICH, even when applied in an unselected ICH population, and in the acute phase of the hemorrhage. This could be beneficial for the speed of the diagnostic process, avoiding delay and inconclusive imaging due to the masking of underlying tumor by hemorrhage.

DECTA aided in the identification of two cases of isolated cortical vein thrombosis, which were not visualized on conventional like CTA (both arterial – and delayed phase imaging). Although CT-venography (CTV), being equivalent to our delayed phase conventional-like CTA, is suitable for detecting cerebral venous sinus thrombosis, it has limited diagnostic value for diagnosing isolated cortical vein thrombosis, having a reported sensitivity of 6–75% [21]. On CTV, congestion of draining vessels surrounding the occluded vein might be the only finding, as the thrombosed vein itself does not opacify. By applying DECTA post processing techniques, such as iodine material decomposition, iodine quantification (to identify a lower iodine load in thrombus) and bone-subtraction, the imaging of isolated cortical vein thrombosis is facilitated, as can be seen in Fig. 2 [22, 23].

For aneurysms, AVMs and DAVFs, the diagnostic yield of DECTA did not differ from that of conventional CTA. In our experience, DECTA nevertheless improves the visuality of these vascular disorders. Other studies reporting on the application of DECT for detecting cerebral vascular malformations are scarce, but suggest a better detection of lesions near osseous structures on DECTA, such as DAVFs and aneurysms. As these studies are performed in a small number of subjects (*N* = 9, *N* = 12 and *N* = 80), encompass primary subarachnoid hemorrhage patients, apply automatic bone removal in three-dimensional images and compare DECTA findings to DSA, comparison to our results is precluded [8, 24, 25].

DVAs, being the most frequently encountered cerebral vascular anomalies in our cohort, are generally considered benign. However, DVAs coexist with cavernous malformations in 13–40% of patients, in which case the combined hemorrhage risk is higher when compared to the hemorrhage risk of cavernous malformations alone [26–28]. DVAs may point to accompanying cavernomas which are often hidden under the hematoma in de the acute phase and are occult on angiography. The finding of a DVAs may thus prompt repeated MRI imaging after resorption of the hematoma. We included DVAs as a possible ICH cause solely when located in, or at the rim of the hematoma.

Despite the large sample size and evaluation of both vascular – and non-vascular secondary ICH causes, our study has limitations. As a consequence of the observational clinical practice design in an unselected ICH population carrying a high mortality, both loss to follow-up and lacking gold standard investigations (often being DSA or MRI/MRA) occurred in a substantial part of included patients. Follow-up was not standardized and thus additional imaging was not performed in every case. False negative findings for both conventional CTA and DECTA are probably underestimated. One can only conclude that the diagnostic yield was higher in DECTA compared to conventional CTA, but sensitivity/specificity analysis cannot be performed. This carries the risk of verification bias and erroneous ICH etiologic classification of patients. Yet, as the primary study goal was to compare the diagnostic yield of conventional CTA to simultaneously performed DECTA in practise, and not to determine diagnostic accuracy, the study design is suitable. Lastly, since the reference standard (final diagnosis at one year) is based on the findings of all examinations during one year follow-up, including data of the conventional-like CTA and DECTA imaging (being the diagnostic tools studied), unavoidably, there is a risk of incorporation bias.

## Conclusion

In this first observational cohort study on the application of DECTA in detecting structural ICH causes, there are strong indications that DECTA is superior to conventional CTA in the early detection of underlying neoplasms, DVAs and CVST in acute ICH, but not for other secondary causes of ICH. Further prospective studies encompassing gold standard and standardized diagnostic workup are advised to evaluate the diagnostic accuracy of DECTA.

## Electronic supplementary material

Below is the link to the electronic supplementary material.

Supplementary Material 1
