# Supplementary figures and images for: Dual-energy CT angiography in detecting underlying causes of intracerebral hemorrhage: an observational cohort study
Source: Neuroradiology. 2024 Oct 25;67(2):331–8. doi: 10.1007/s00234-024-03473-1 (PMC11893716; doi:10.1007/s00234-024-03473-1)

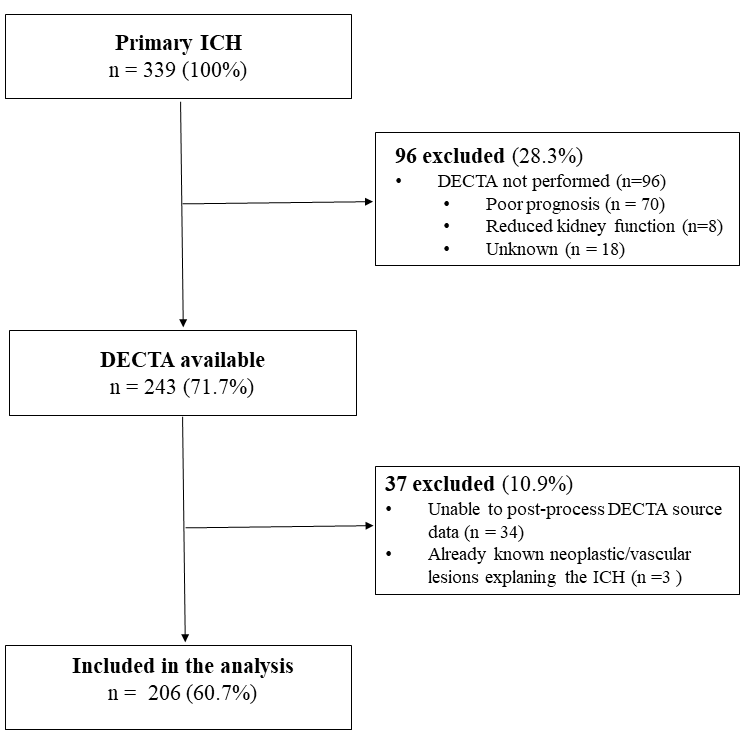

Supplement: Supplementary file 1 — Supplementary Material 1 [file 234_2024_3473_MOESM1_ESM.tif]
